# Liver fat scores do not reflect interventional changes in liver fat content induced by high-protein diets

**DOI:** 10.1038/s41598-021-87360-2

**Published:** 2021-04-23

**Authors:** Stefan Kabisch, Mariya Markova, Silke Hornemann, Stephanie Sucher, Olga Pivovarova-Ramich, Jürgen Machann, Johannes Hierholzer, Sascha Rohn, Andreas F. H. Pfeiffer

**Affiliations:** 1 Department of Clinical Nutrition, German Institute of Human Nutrition Potsdam-Rehbrücke, Arthur-Scheunert-Allee 114-116, 14558 Nuthetal, Germany; 2 German Center for Diabetes Research (Deutsches Zentrum Für Diabetesforschung e.V.), Ingolstädter Landstraße 1, 85764 Neuherberg, Germany; 3 Department of Endocrinology, Diabetes and Nutrition, Charité University Medicine, Campus Benjamin Franklin, Hindenburgdamm 30, 12203 Berlin, Germany; 4 Research Group Molecular Nutritional Medicine, Department of Molecular Toxicology, German Institute of Human Nutrition Potsdam-Rehbrücke, Nuthetal, Germany; 5 Institute of Diabetes Research and Metabolic Diseases of the Helmholtz Center Munich at the University of Tübingen, Tübingen, Germany; 6 Section on Experimental Radiology, Department of Diagnostic and Interventional Radiology, University Hospital Tübingen, Tübingen, Germany; 7 Department of Diagnostic and Interventional Radiology, Ernst von Bergmann Hospital, Potsdam, Germany; 8 Institute for Food and Environmental Research, Nuthetal, Germany; 9 Institute of Food Chemistry, Hamburg School of Food Science, University of Hamburg, Hamburg, Germany; 10 Department of Food Chemistry and Analysis, Institute of Food Technology and Food Chemistry, Technische Universität Berlin, TIB 4/3-1, Gustav-Meyer-Allee 25, 13355 Berlin, Germany

**Keywords:** Endocrine system and metabolic diseases, Gastrointestinal diseases, Metabolic disorders, Nutrition disorders

## Abstract

Non-alcoholic fatty liver disease (NAFLD) is common in Metabolic Syndrome and type 2 diabetes (T2DM), driven by energy imbalance, saturated fats and simple carbohydrates. NAFLD requires screening and monitoring for late complications. Liver fat indices may predict NAFLD avoiding expensive or invasive gold-standard methods, but they are poorly validated for use in interventional settings. Recent data indicate a particular insensitivity to weight-independent liver fat reduction. We evaluated 31 T2DM patients, completing a randomized intervention study on isocaloric high-protein diets. We assessed anthropometric measures, intrahepatic lipid (IHL) content and serum liver enzymes, allowing AUROC calculations as well as cross-sectional and longitudinal Spearman correlations between the fatty liver index, the NAFLD-liver fat score, the Hepatosteatosis Index, and IHL. At baseline, all indices predicted NAFLD with moderate accuracy (AUROC 0.731–0.770), supported by correlation analyses. Diet-induced IHL changes weakly correlated with changes of waist circumference, but no other index component or the indices themselves. Liver fat indices may help to easily detect NAFLD, allowing cost-effective allocation of further diagnostics to patients at high risk. IHL reduction by weight-independent diets is not reflected by a proportional change in liver fat scores. Further research on the development of treatment-sensitive indices is required.

Trial registration: The trial was registered at clinicaltrials.gov: NCT02402985.

Non-alcoholic fatty liver disease (NAFLD) is a common metabolic disorder with increasing prevalence^1^. Paralleling its epidemiological development with type 2 diabetes (T2DM), both manifestations of the Metabolic Syndrome are mainly driven by a low energy expenditure and non-proportionally high energy intake^2^.

Hepatosteatosis may progress further to non-alcoholic steatohepatitis (NASH), hepatic fibrosis, hepatic cirrhosis, and primary hepatic malignoma, highlighting the need for prevention and therapy^3^.

To detect early NAFLD, ultrasound sonography may be helpful, but requires experienced examining personnel and good imaging quality^4^. Sonoelastography can amplify a first diagnosis by adding information about liver tissue density, but not all causes of liver fibrosis are necessarily linked to high liver fat content allowing misinterpretation^5,6^. Computed tomography (CT) and magnetic resonance-(MR)-based techniques are expensive techniques and either accommodated by x-ray exposure or contraindications to due magnetic fields. Both, CT and MRI (before the era of multi-echo Dixon assessment of proton density fat fraction) provide an only moderate sensitivity and specificity for NAFLD detection and graduation^7^.

On the other hand, proton MR spectroscopy (^1^H-MRS) indeed measures tissue lipid content, but is mainly used in clinical studies due to high instrumental costs. Liver biopsies provide a highly specific measurement of intrahepatic lipid (IHL) amount, but are prone to misinterpretation in cases of heterogeneous fat distribution. Also, there is a significant risk of liver injury, bleeding, bile leakage and further complications.

To assess and monitor NAFLD with low costs in every subject at risk, several NAFLD scores have been developed. The fatty liver index (FLI) uses markers of obesity (body mass index (BMI) and waist circumference), dyslipidemia (triglycerides (TG)), and liver injury (gamma-glutamyltransferase (GGT) and correlates with liver fat content, predicting the majority of NAFLD cases^8^. However, waist circumference and visceral obesity seem to be the main factors in its prediction models^9,10^. The NAFLD-liver fat score (NAFLD-LFS) requires assessment of fasting insulin and transaminases as well as the subjects’ classification regarding the Metabolic Syndrome. The prediction quality of the NAFLD-LFS is comparable to the FLI^11^ and both indices are designed and validated for Caucasian populations. Genetic markers such as P184L in the Patatin-Like Phospholipase Domain-Containing Protein 3 (PNPLA3) gene have been shown to improve their power^11,12^. In Asian cohorts, the hepatic steatosis index (HSI) was demonstrated as a suitable NAFLD prediction tool^13^, but ethnicity appears to be a relevant factor of those prediction models^14^. This led to the development of some more indices in the past, focusing on various ethnicities^15–17^.

These three indices have been shown to be useful in a population-wide perspective in order to estimate NAFLD-associated mortality^18^.

All NAFLD scores are only moderate in sensitivity and specificity, with respect to the also-flawed sonography^19^ and under validation by biopsy^20^. NAFLD in children is possibly easier to predict, as adiposity and metabolic disorder are more purely involved in disease progression in this sub-group, excluding ageing and long-term toxicity of specific environmental factors^21,22^. Sonography examination is highly dependent on examiner’s expertise and ranks inferior to liver fat indices^23^. Also, no serum marker has been demonstrated to outrank conventional liver fat indices^24,25^.

As an important factor, sex affects some components of NAFLD indices, implying differences in the predictive quality for men and women^26,27^. However, only the HSI accounts for these differences^13^.

Additionally, HSI and FLI seem to fail to sufficiently predict NAFLD in subjects with overt T2DM, possibly due to interactions with typical medications in these patients, wide-range daily fluctuations of fasting glucose or lower discriminative range of the HSI. This is plausible, as literally all subjects with overt T2DM fulfill a major criterion of the index with no regard to their liver status^13^.

Furthermore, there is almost no published data on the value of NAFLD indices in longitudinal settings. It is unclear, whether changes in NAFLD scores mirror changes in actual liver fat content. Lipid parameters are not yet sufficiently investigated to be of use during the monitoring process^21^. NAFLD-LFS and FLI showed an only weak to moderate performance as monitoring tools within low-fat lifestyle intervention trials^12,28,29^. On the other hand, their usefulness within a low-carb diet could not be shown^29^. Missing correlation between change of liver fat indices and change of actual IHL was attributed to the missing correlation between weight change and liver fat reduction. Similar effects need to be expected from glitazones, but also from dietary approaches with poly-unsatured fatty acids, which act independently of weight loss^30^. A recent short-term trial on high-protein diets has demonstrated, that this approach is another way to reduce liver fat without relevant loss in body weight^31,32^.

As liver fat reduction does not require weight loss, more data is needed to evaluate, if liver fat scores can reflect changes of IHL content independently of the therapeutic approach.

Therefore, we investigate the statistical relation between changes in three liver fat scores in a human lifestyle intervention trial, featuring two isocaloric diets. For our analysis, assessments of liver fat content by ^1^H-MRS and liver fat scores on the basis of anthropometric measurements and fasted blood samples are available.

## Methods

Data for this publication are extracted from the lifestyle intervention trial, registered at clinicaltrials.gov: NCT02402985 (submitted on 4th February 2015, first posted on 31st March 2015). This randomized parallel-designed trial compared two isocaloric six-week dietary interventions with either plant- or an animal-based high-protein diet in subjects with T2DM. The study was conducted in accordance with the Declaration of Helsinki. The ethics committee of the University of Potsdam approved the study protocol. Recruitment for this study started in September 2013 and was completed in March 2015. All subjects provided their informed consent prior to participation. Data from the trial, including a detailed section about the study protocol, was already published elsewhere^31,32^.

At baseline, the participants of the study underwent fasting blood sampling, a mixed meal tolerance test, full anthropometry (body weight, height, abdominal circumferences, bio-impedance analysis) and medical examination. Study volunteers were also subjected to liver ^1^H-MRS on a 1.5 T whole body imager (Magnetom Avanto, Siemens Healthcare, Erlangen, Germany) according to pre-published specifications^33^. In brief, a single voxel STimulated Echo Acquisition Mode (STEAM) technique with short echo time (TE = 10 ms) and long repetition time (TR = 4 s) was applied and the volume of interest (30 × 30 × 20 mm^3^) was placed in the posterior part of segment 7. Ratio of the integral of methylene and methyl resonances (lipids) and water + lipids was calculated to determine IHL.

Data from this publication is restricted to subjects with complete data sets on liver fat content and liver fat scores (per-protocol analysis on diet phase completers with no respect to dietary in-/compliance). Subjects with severe cardiopulmonary, hepatic, metabolic, psychiatric, infectious or inflammatory disease are excluded from the entire study. Also, participants with increased ethanol intake (men: above 30 g per day; women: above 20 g per day) are not part of the presented data set.

After screening, inclusion and baseline assessment with ^1^H-MRS, subjects were 1:1-randomised to one of two dietary regimes with comparable isocaloric nutrient distribution of 25–30% relative energy intake (EI%) of protein, 40 EI% of carbohydrates, 30–35 EI% of fat, but differing in their protein source: animal protein (dairy/meat) or legumes. Randomisation was done by non-clinical personnel by group matching for age, sex, body mass index, hemoglobin A1c, and glucose-lowering drugs using a random number generator. Dietary intervention (supplementation with neutrally labelled high-protein food products of similar appearance) was conducted for 6 weeks. All assessments from the baseline visit were repeated after that time period.

Subjects were instructed not to change physical activity.

Animal and plant protein diet showed a comparable outcome with respect to overall performance including liver fat reduction, both diet groups were treated as combined cohort for this analysis^31,32^.

Statistical analysis entails calculation of area-under-the-responder-operator-curves (AUROC) for the prediction of NAFLD by FLI, NAFLD-LFS and HSI at baseline. FLI, NAFLD-LFS and HSI were calculated according to their first publication^8,11,13^. Also, Spearman correlations were used to evaluate cross-sectional prediction of NAFLD at baseline and longitudinal monitoring of NAFLD during the diet phase.

Additional correlation analyses were performed to elucidate potential reasons or mechanisms for diet-dependent results. SPSS 25.0 was used for all statistical calculations.

## Results

31 subjects of the study with full data on IHL and liver fat indices were selected for the presented data set. Baseline characteristics for the cohort are presented in Table 1.

**Table 1:** Baseline characteristics.

Parameter	(n = 31)
Age (years)	65 ± 6
Sex (male/female)	19 m/12 f
Liver fat content (MR-S; %)	15.4 ± 9.8
FLI	74 ± 23
NAFLD-LFS	0.54 ± 1.16
HSI	40 ± 4
Body weight (kg)	89.4 ± 14.2
BMI (kg/m^2^)	30.6 ± 3.7
Waist circumference (cm)	102.9 ± 10.9
Fasting insulin (mU/L)	9.48 ± 6.29
Triglycerides (mmol/L)	1.70 ± 0.59
AST (U/ml)	25 ± 9
ALT (U/ml)	28 ± 10
AST/ALT ratio	0.9 ± 0.2
GGT (U/ml)	44 ± 26

*ALT* alanine aminotransferase, *AST* aspartate aminotransferase, *GGT* gamma-glutamyltransferase, *FLI* fatty liver index, *HSI* Hepatosteatosis Index, *NAFLD-LFS* Non-alcoholic fatty liver disease-liver fat score; no significant differences between both groups.

### NAFLD prediction and correlation at baseline

**Figure 1:**
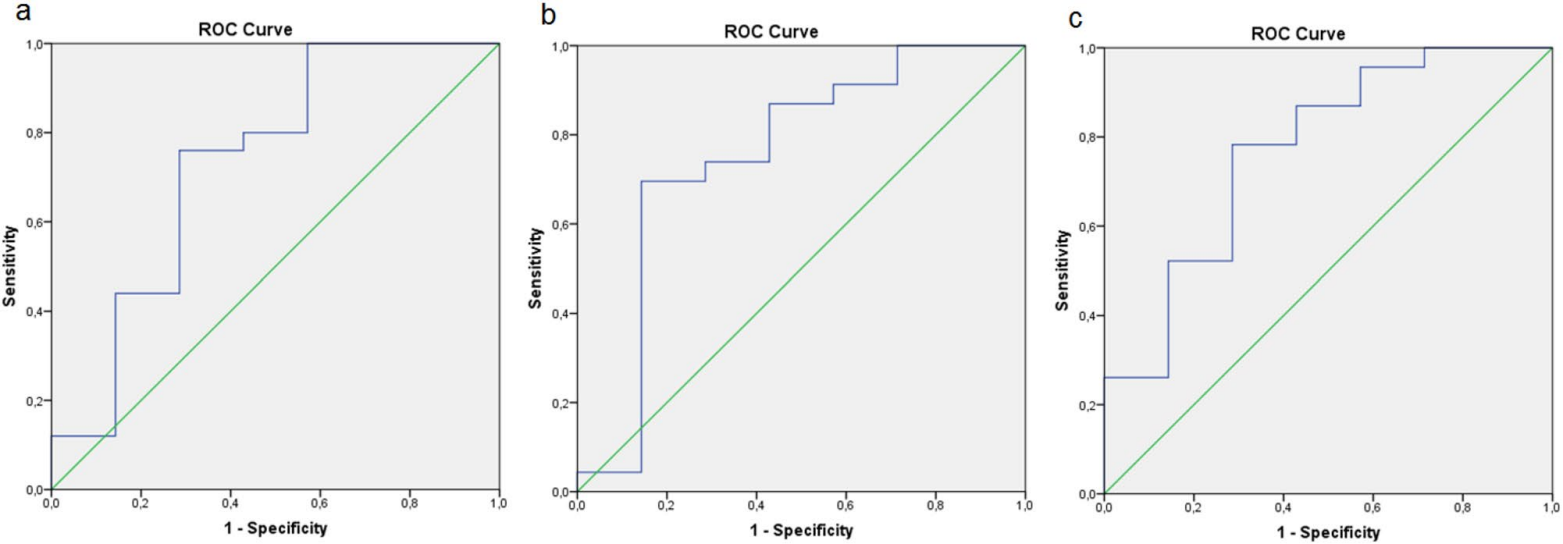
AUROC representation of NAFLD prediction by FLI (**a**), NAFLD-LFS (**b**) and HSI (**c**); the analyses show AUROC values of 0.731 (FLI), 0.752 (NAFLD-LFS) and 0.770 (HSI).

**Figure 2:**
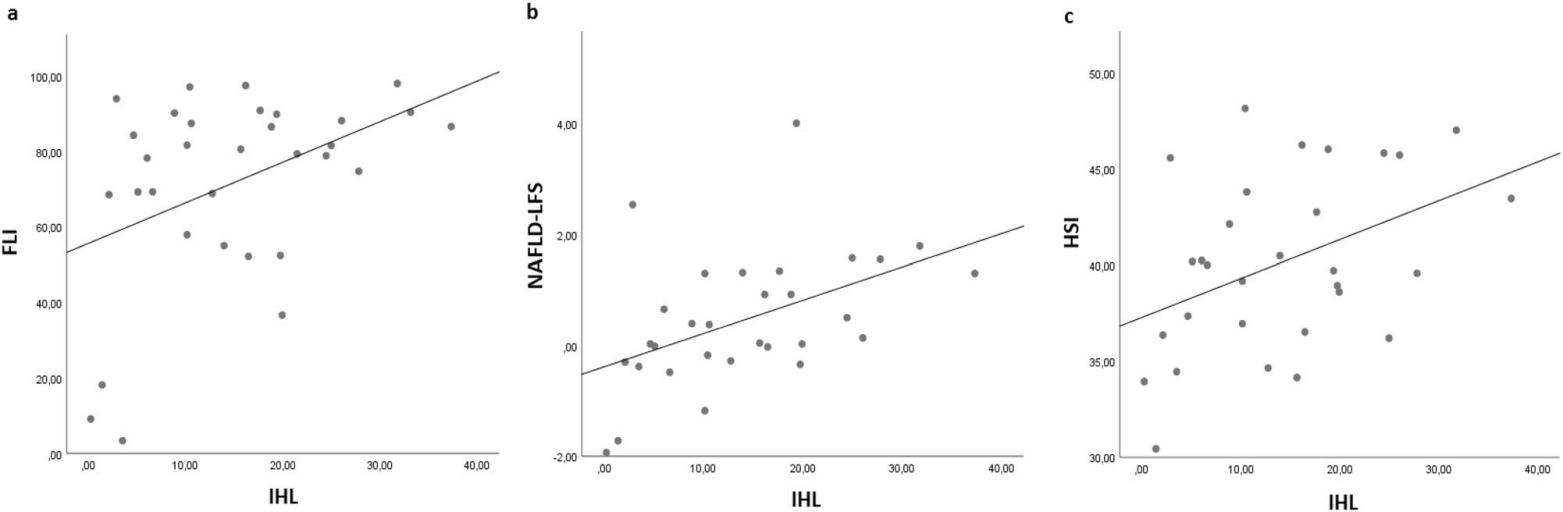
Correlation analysis between liver fat indices and IHL at baseline; FLI = fatty liver index, HSI = Hepatosteatosis Index, IHL = intrahepatic lipids, NAFLD-LFS = non-alcoholic fatty liver disease-liver fat score; (**a**) IHL ~ FLI; rho = 0.351. *p* = 0.049; (**b**) IHL ~ NAFLD-LFS; rho = 0.537, *p* = 0.002; (**c**) IHL ~ HSI; rho = 0.393, *p* = 0.032.

NAFLD prediction by FLI, NAFLD-LFS and HSI is comparable to their first publication^8,11,13^, resulting in AUROC values of 0.731 for FLI, 0.752 for NAFLD-LFS and 0.770 for HSI in the entire cohort, respectively (Fig. 1A–C). Accordingly, correlations between IHL and each of the three liver fat indices were significant at baseline (Fig. 2A–C).

### ***Correlations between change of index values and interventional ***^***1***^***H-MRS data***

**Figure 3:**
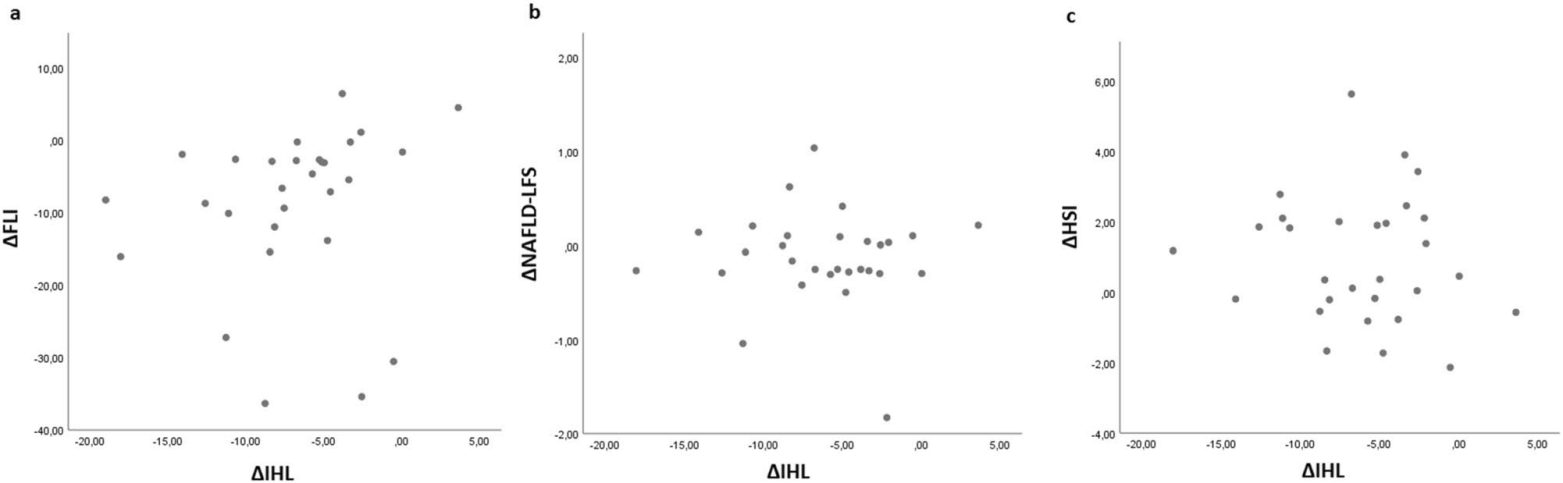
Correlation analysis with changes of liver fat indices and IHL; FLI = fatty liver index, HSI = Hepatosteatosis Index, IHL = intrahepatic lipids, NAFLD-LFS = non-alcoholic fatty liver disease-liver fat score; (**a**) IHL ~ FLI; rho = 0.342. *p* = 0.069; (**b**) IHL ~ NAFLD-LFS; rho =  − 0,058, *p* = 0.765; (**c**) IHL ~ HSI; rho =  − 0.049, *p* = 0.800.

As reported in our previous publication, liver fat reduction in these 31 subjects was very strong (6.7 ± 5.0%-pts.), but was hardly explained by the very limited weight loss of 2.1 ± 1.7 kg^31^. In this interventional perspective there was no significant correlation between changes of IHL and changes of NAFLD-LFS or HSI, but we report a weak, trendwise association between change of IHL and change of FLI. (Fig. 3).

### ***Correlations between changes of index parameters and ***^***1***^***H-MRS based IHL data***

Correlation analysis between change of actual IHL content and change of single elements of the liver fat scores revealed just one significant correlation—for the reduction of waist circumference. The similarly weak correlation with weight loss failed to achieve statistical significance (Table 2).

**Table 2:** Correlation analysis on interventional changes.

Correlation between change of liver fat content (MR-S) and change of …	(n = 31)
Body weight (kg)	Rho = 0.347, *p* = 0.056
Waist circumference (cm)	Rho = 0.409, *p* = 0.022 *
Fasting insulin (pmol/L)	Rho = − 0.026, *p* = 0.890
Triglycerides (mg/dl)	Rho = 0.299, *p* = 0.103
AST (U/ml)	Rho = 0.091, *p* = 0.626
ALT (U/ml)	Rho = 0.200, *p* = 0.298
AST/ALT ratio	Rho = − 0.133, *p* = 0.490
GGT (U/ml)	Rho = 0.219, *p* = 0.254

*ALT* alanine aminotransferase, *AST* aspartate aminotransferase, *GGT* gamma-glutamyltransferase; *p < 0.05; **p < 0.01; ***p < 0.001.

Splitting any of the above-mentioned analyses by diet group did not lead to numerically different results (data not shown).

## Discussion

Liver fat scores are an easy and cheap tool to assess NAFLD status in metabolic research, but also in clinical practice. These tools might save time, monetary personal and technical resources, as they are suitable for any kind of patient and do not require expensive body imaging.

However, previous data on the prediction quality of these scores show a limited predictive performance^34^. Within the present study, there is additional support for the use of these indices in cross-sectional settings.

Similar to a recent publication on a hypocaloric high-protein low-carb diet, the current analysis in subjects undergoing an isocaloric high-protein diet also reveals poor performance in a longitudinal approach^29^. Liver fat reduction by this particular diet, independently of weight loss, is not reflected by any liver fat score with satisfying precision. IHL reduction in low-fat diets—with a consistent dependency of metabolic improvement on weight loss—seems to be the only dietary NAFLD treatment, that can be monitored by liver fat scores^12,28^.

By correlation analysis we showed in our T2DM sample, that combined liver fat scores do not sufficiently reflect the actual change in IHL. While the FLI may provide a weak correlation in a larger data set, both NAFLD-LFS and HSI completely failed to mirror the changes in liver fat content. Apparently, the FLI outranks these indices as all FLI elements—body weight, waist circumference, triglycerides and GGT—are those single parameters which are most strongly connected to change of IHL. Among all liver enzymes, GGT is the most sensitive liver parameter for prediction of NAFLD and its sequelae^35–37^ Insulin levels—being part of the badly performing NAFLD-LFS—may not necessarily reflect a metabolic improvement, especially in the context of T2DM patients with primarily reduced insulin secretion capacity, who often lack NAFLD^11,38^. The HSI was developed for patients of Asian ethnicity, maybe limiting its application to Caucasian patients^13^.

Most individual index parameters do not correlate with ^1^H-MRS derived change of liver fat. The strongest connection between change of liver fat and other single anthropometabolic outcomes was seen with waist circumference. Apparently, in our high-protein setting, both visceral and hepatic fat depots are reduced simultaneously^31^. However, change in body weight does not relevantly correlate with change in IHL in the present cohort. As measures of obesity are a major component of all liver fat scores, limited weight loss might be the crucial factor in missing correlation between change of liver fat scores and change of IHL. In our data set of the isocaloric LeguAN study, we observed a rather minor weight loss of 2.1 ± 1.7 kg over an intervention period of six weeks. Most possibly, the same finding as ours could be present in isocaloric diets with improved dietary fat composition or in pharmaceutical treatments (glitazones, gliflozines), which have been shown to reduce liver fat independently of weight loss. As a strength of our analysis, the observed liver fat reduction is marked, especially given the very moderate weight loss. Thus, we can clearly show the dissociation of adiposity and NAFLD as metabolic outcomes of the study, but also as aspects of liver fat indices.

In comparison to a recently published analysis on IHL monitoring in a low-carb diet, correlations with several metabolic parameters in our cohort were at least similar in direction, if not magnitude. Especially changes in ALT and IHL reduction correlated under low-carb, but not low-fat conditions^29^. High-protein diets seem to beneficially affect lipid levels, including triglycerides, and all transaminase levels. Missing correlation between these changes and change of IHL might indicate additional actions beyond mere steatosis. Possibly, transaminase levels in these patients do also reflect a certain degree of inflammation (NASH) or fibrosis, which is improved in parallel. The possibility of dietary effects even on high-stage fibrosis has been discussed recently^39^.

New prediction scores might include parameters with higher prediction quality and specificity, such as ferritin^40^.

Some limitations have to be addressed for the present study. Our data set provides a moderate statistical power compared to other studies on this issue. However, it is the first publication on subjects with overt T2DM.

Further, we are unable to fully clarify the reasons for missing comparability of longitudinal correlations between liver fat scores and MR-based liver fat values in different diets. As shown in recent publications, liver fat reduction can be achieved in accompaniment of highly variable metabolic improvements, depending on type of diet. This diet-specific interaction also entails linkage of IHL reduction and total weight loss in low-fat diets, but not low-carb or high-protein diets^29^.

Conclusively, with the present study, we underline the good predictive properties of FLI, NAFLD-LFS and HSI before a dietary intervention in subjects with overt T2DM by both AUROC analysis and Spearman correlation. However, liver fat reduction is not accompanied by correlating liver fat scores.

Liver fat indices need to be used cautiously, especially when assessing changes in different interventional settings. More research is needed to elaborate effects of specific diets on metabolic components and blood parameters that could be used for the design of a liver fat index.

## Abbreviations

ALT: Alanine aminotransferase
AST: Aspartate aminotransferase
BMBF: Bundesministerium für Bildung und Forschung (German Federal Ministry for Education and Research)
BMEL: Bundesministerium für Ernährung und Landwirtschaft (German Federal Ministry for Food and Agriculture)
BMI: Body-mass index
CT: Computed tomography
DZD: Deutsches Zentrum für Diabetesforschung (German Center for Diabetes Research)
EI: Energy intake
FLI: Fatty liver index
GGT: Gamma-glutamyltransferase
HSI: Hepatic steatosis index
IHL: Intrahepatic lipids
MRS: Magnetic resonance spectroscopy
NAFLD: Non-alcoholic fatty liver disease
NAFLD-LFS: NAFLD-liver fat score
NASH: Non-alcoholic steatohepatitis
PNPLA3: Patatin-like phospholipase domain-containing protein 3
STEAM: STimulated echo acquisition mode
T2DM: Type 2 diabetes mellitus
TG: Triglycerides
